# The Journal in the Horizon of Asia and Europe

**Published:** 2018-05-01

**Authors:** Sheikh Mohammad Fazle Akbar, Hasan Ozkan, Mamun Al-Mahtab

**Affiliations:** 1 Editor-in-Chief, Euroasian Journal of Hepato-Gastroenterology; 2 Editor-in-Chief, Euroasian Journal of Hepato-Gastroenterology; 3 Co-Editor-in-Chief, Euroasian Journal of Hepato-Gastroenterology

The editors, members of the editorial board, publishers, reviewers, authors, and all other entities related to the Euroasian Journal of Hepato-Gastroenterology are extremely happy as the articles of the journal are now referred in PubMed. This is in addition to its indexing in several other indexing organizations. Our thanks and gratitude are for all who have been working for this important endorsement and those who worked for this purpose.

Indexing of journals in PubMed is not the only and final target of the editors and publisher. The journal should be placed in better position by having an impact factor within a reasonable time. To achieve this goal, some fundamental changes would be incorporated soon.

The journal will act as an official journal of the Euroasian Gastroenterological Association (EGA). A strong executive committee of EGA would constantly look after the journal.

The editorial board of the journal will be strengthened and formulated to meet the demand of the time. The instructions to authors will be made more objective-oriented and the authors will get better review process for their precious article.

The numbers of issues of the journal will be extended from the present levels of two per year. All these efforts would finally make this journal one of the most prestigious journals in this region. We all are expecting your active cooperation to accomplish this goal.

We are extremely glad of the work and collaboration of the publisher of the journal, JAYPEE Publisher, for their outstanding support in the last 7 years and hope that these scholastic and materialistic support would be provided for years to come.

Finally, we would remind that a journal is an outcome of a team work and we do hereby express our gratitude to all seen and unseen entities related to journal compilation and publication.

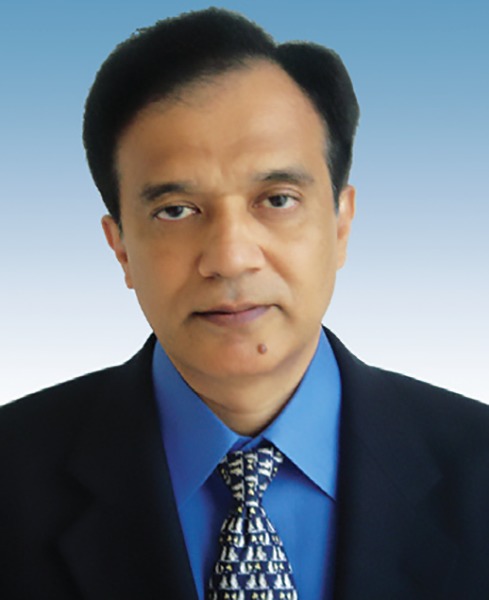



**Sheikh Mohammad Fazle Akbar**

Editor-in-Chief

Euroasian Journal of Hepato-Gastroenterology

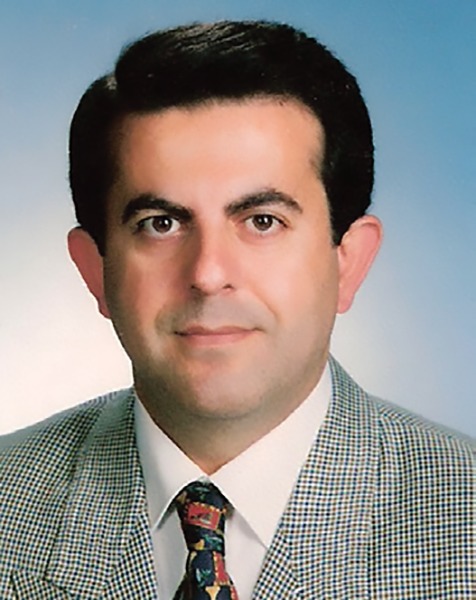



**Hasan Ozkan**

Editor-in-Chief

Euroasian Journal of Hepato-Gastroenterology

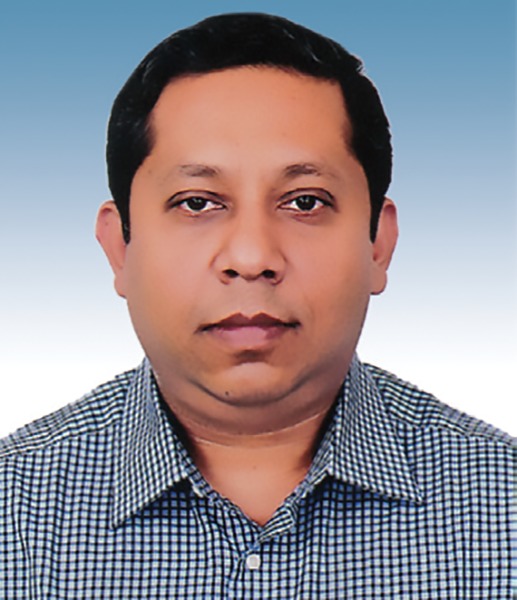



**Mamun Al-Mahtab**

Co-Editor-in-Chief

Euroasian Journal of Hepato-Gastroenterology

